# Ultrathin silicon oxynitride layer on GaN for dangling-bond-free GaN/insulator interface

**DOI:** 10.1038/s41598-018-19283-4

**Published:** 2018-01-23

**Authors:** Kengo Nishio, Tomoe Yayama, Takehide Miyazaki, Noriyuki Taoka, Mitsuaki Shimizu

**Affiliations:** National Institute of Advanced Industrial Science and Technology (AIST), Central 2, Umezono 1-1-1, Tsukuba, Ibaraki 305-8568 Japan

## Abstract

Despite the scientific and technological importance of removing interface dangling bonds, even an ideal model of a dangling-bond-free interface between GaN and an insulator has not been known. The formation of an atomically thin ordered buffer layer between crystalline GaN and amorphous SiO_2_ would be a key to synthesize a dangling-bond-free GaN/SiO_2_ interface. Here, we predict that a silicon oxynitride (Si_4_O_5_N_3_) layer can epitaxially grow on a GaN(0001) surface without creating dangling bonds at the interface. Our *ab initio* calculations show that the GaN/Si_4_O_5_N_3_ structure is more stable than silicon-oxide-terminated GaN(0001) surfaces. The electronic properties of the GaN/Si_4_O_5_N_3_ structure can be tuned by modifying the chemical components near the interface. We also propose a possible approach to experimentally synthesize the GaN/Si_4_O_5_N_3_ structure.

## Introduction

When two different materials are joined together, the interface is formed. Since the atomic structure of the interface has a significant impact on its electronic properties, understanding and controlling the interfaces at atomistic level has been a challenge of materials science. Particularly, forming high-quality interfaces is important in a wide range of applications. For example, the success of Si-based metal-insulator-semiconductor field-effect transistors (MISFETs) is largely relied on the formation of the atomically-abrupt and low-dangling-bond-density interface between silicon (Si) and silicon dioxide (SiO_2_)^1–5^.

Recently, gallium nitride (GaN) has attracted much attention for applications in power electronic devices^6^. Since the dangling bonds formed at the GaN/insulator interface create trap states which degrade carrier mobility, reducing the interface dangling bonds is a key to improve the performance of GaN-based MISFETs^5,7–11^.

Given that knowledge of SiO_2_ has been accumulated in developing Si-based MISFETs, SiO_2_ is one of choices for the insulator of GaN-based MISFETs^5,8–11^. However, since the atomic structure of SiO_2_ is different from that of GaN, dangling bonds are easily formed at the GaN/SiO_2_ interface. To overcome this problem, the mechanism which allows for the formation of the high-quality Si/SiO_2_ interface would be helpful. Although bulk crystalline SiO_2_ cannot grow epitaxially on crystalline Si due to the lattice mismatch, an atomically-thin ordered layer can form between crystalline Si and amorphous SiO_2_, serving as a buffer for smoothly connecting the different structures^1–4^. Therefore, the formation of a buffer layer between crystalline GaN and amorphous SiO_2_ would be a key to synthesize a high-quality GaN/SiO_2_ interface^12^.

Since GaN has a small lattice mismatch with SiC, and both of GaN and SiC consist of honeycomb layers, knowledge of silicon carbide (SiC)^13–20^ would also be helpful in searching for an ideal GaN/SiO_2_ structure. It is known that, although the lattice mismatch prevents the epitaxial growth of bulk crystalline SiO_2_ on crystalline SiC, it is possible to epitaxially grow atomically-thin silicon-oxide layers on surfaces of crystalline SiC^13,16^. Specifically, when a SiC(0001) surface is etched in hydrogen, an epitaxial Si_2_O_3_ layer grows on the SiC(0001) surface. On the other hand, when a SiC(000 $\bar{1}$) surface is etched in hydrogen, an epitaxial Si_2_O_5_ layer grows on the SiC(000 $\bar{1}$) surface. Although the SiC/Si_2_O_3_ and SiC/Si_2_O_5_ structures both have atomically-abrupt interfaces, dangling bonds remain at the interfaces. As a result, dangling bond states exist within the band gap of the SiC/Si_2_O_3_ and SiC/Si_2_O_5_ structures^14,15^. On the other hand, when the nitrogen treatment is performed after the hydrogen-gas etching, an epitaxial Si_4_O_5_N_3_ layer grows on a SiC(0001) surface^16^. Interestingly, no dangling-bond states exist within the band gap of the SiC/Si_4_O_5_N_3_ structure because the dangling bonds of Si atoms on the clean SiC surface, as well as those of the Si_4_O_5_N_3_ layer, are all terminated^16–19^. The SiC/Si_4_O_5_N_3_ structure is therefore expected to be a seed for a high-quality SiC/SiO_2_ structure^20^.

The GaN/Si_4_O_5_N_3_ structure has not been synthesized experimentally yet. However, resting on the experimentally synthesized SiC/Si_4_O_5_N_3_ structure and the small lattice mismatch between GaN and SiC, we argue that the Si_4_O_5_N_3_ layer can epitaxially grow on a GaN(0001) surface. To our knowledge, however, such a possibility has not been recognized so far. Recently, the impact of computer simulations to offer guidelines in the identification of potentially useful new materials has been increasing^21–24^. For example, silicene was first predicted by *ab initio* calculations^21^ and then later on synthesized experimentally^22,23^. In this paper, being inspired by the geometrical analogies between SiC and GaN and the experimentally synthesized SiC/Si_4_O_5_N_3_ structure, we investigate the stability of the GaN/Si_4_O_5_N_3_ structure using *ab initio* calculations, and propose that the GaN/Si_4_O_5_N_3_ structure is experimentally accessible. We also study the band structure of the GaN/Si_4_O_5_N_3_ structure and its derivatives.

## Results and Discussions

### Atomic structure and stability of the GaN/Si_4_O_5_N_3_ structure

**Figure 1:**
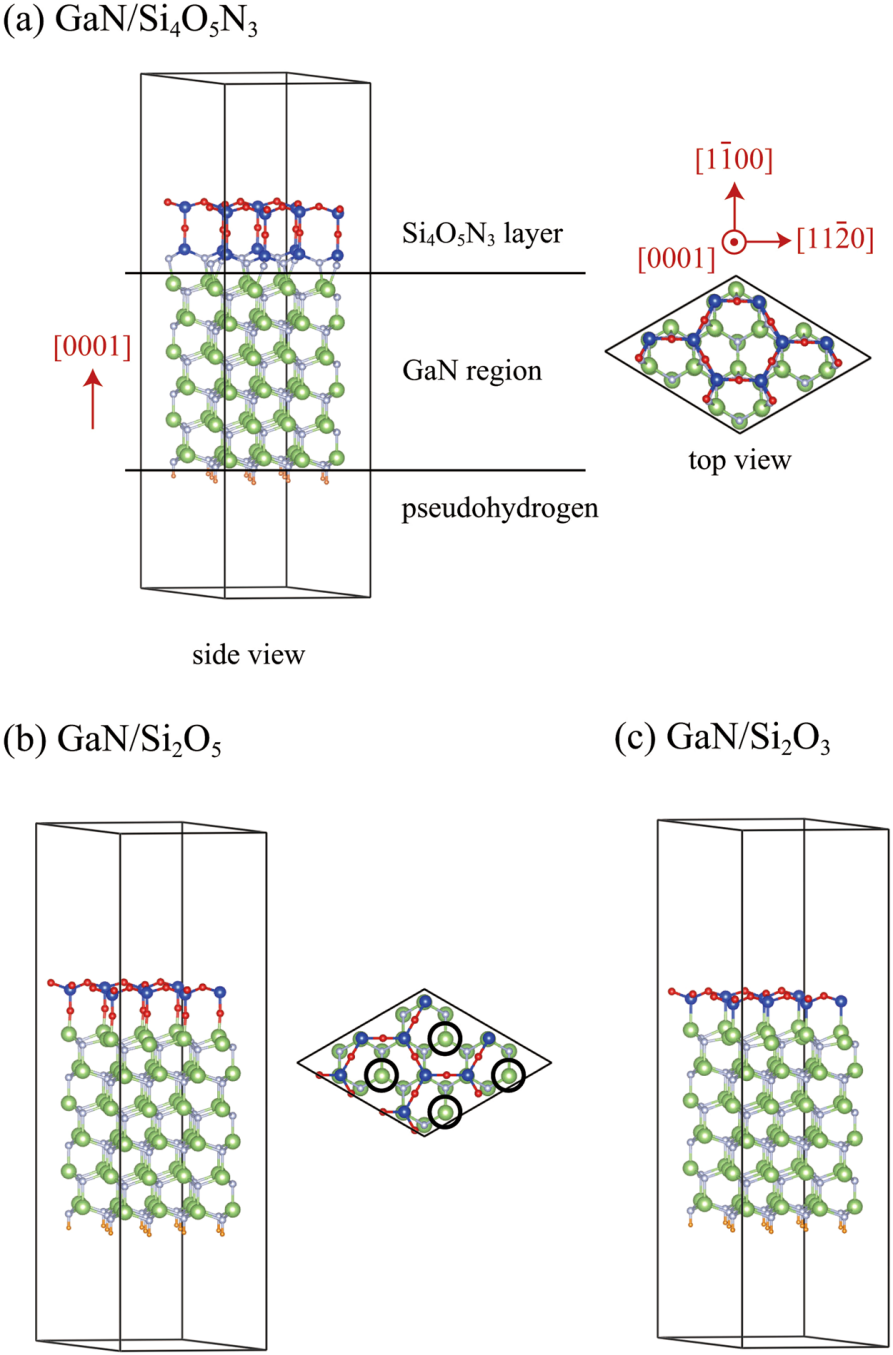
Structure model. (**a**) GaN/Si_4_O_5_N_3_ structure. Red, blue, white, green, and orange spheres represent O, Si, N, Ga, and pseudohydrogen atoms, respectively. (**b**) GaN/Si_2_O_5_ structure. Each circled Ga atom has a dangling bond. (**c**) GaN/Si_2_O_3_ structure.

Figure 1a illustrates the optimized structure model of the GaN/Si_4_O_5_N_3_ structure. All the dangling bonds of the Ga atoms at the GaN(0001) surface are terminated by the N atoms of the Si_4_O_5_N_3_ layer. We refer to the Ga atoms bonded to the N atoms of the Si_4_O_5_N_3_ layer as interface Ga atoms. Similarly, we refer to the N atoms of the Si_4_O_5_N_3_ layer bonded to the interface Ga atoms as interface N atoms. As with Ga atoms of GaN, each interface Ga atom of the GaN/Si_4_O_5_N_3_ structure is bonded to four N atoms: one interface N atom of the Si_4_O_5_N_3_ layer and three N atoms of GaN. However, because the interface N atoms of the Si_4_O_5_N_3_ layer are not located right above the interface Ga atoms, the tetrahedra formed by the N atoms surrounding the interface Ga atoms are distorted. Unlike the N atoms of GaN, each interface N atom of the Si_4_O_5_N_3_ layer is bonded to three neighbouring atoms: one interface Ga atom and two Si atoms of the Si_4_O_5_N_3_ layer. Thus, the chemical environments around the interface Ga and N atoms are different from those of Ga and N atoms of GaN, respectively.

For comparison, the GaN/Si_2_O_5_ structure is illustrated in Fig. 1b. The Si_2_O_5_ layer is obtained from the Si_4_O_5_N_3_ layer by removing Si_2_N_3_ (all the N atoms and Si atoms bonded to those N atoms). The formation energy per Si_2_N_3_ of the GaN/Si_4_O_5_N_3_ structure relative to the GaN/Si_2_O_5_ structure is 17.4 eV. The positive value indicates that the dangling bond free GaN/Si_4_O_5_N_3_ structure is energetically more stable than the GaN/Si_2_O_5_ structure. Note that the O atoms of the Si_2_O_5_ layer are bonded to the Ga atoms of the GaN(0001) surface. However, four surface Ga atoms are not bonded to the Si_2_O_5_ layer, in other words, the GaN/Si_2_O_5_ structure has four interface dangling bonds per surface supercell.

We also illustrate the GaN/Si_2_O_3_ structure in Fig. 1c. This structure is obtained from the GaN/Si_2_O_5_ structure by removing O atoms which bridge Ga atoms and Si atoms, and then directly connecting the Si atoms to the Ga atoms. The formation energy per O atom of the GaN/Si_2_O_3_ structure relative to the GaN/Si_2_O_5_ structure is −3.95 eV. The negative value indicates that the GaN/Si_2_O_5_ structure is energetically more stable than the GaN/Si_2_O_3_ structure.

The GaN/Si_4_O_5_N_3_ structure is more stable than the GaN/Si_2_O_5_ structure which is more stable than the GaN/Si_2_O_3_ structure. Thus, the GaN/Si_4_O_5_N_3_ structure is most stable of three. To further examine the stability of the GaN/Si_4_O_5_N_3_ structure, we carried out 2-picosecond molecular dynamics simulations at 1000 K. The GaN/Si_4_O_5_N_3_ structure endured the stability test. Note that the ttt1, tttt1, and SiC-inserted structures, which will be discussed later, also endured the stability test.

### Clue to the realization of our proposal

Our results given above suggest that the GaN/Si_4_O_5_N_3_ structure is experimentally accessible. Here, we propose an approach to realize it. For this purpose, we first point out that the SiC/Si_4_O_5_N_3_ structure is formed by the nitrogen treatment followed by the hydrogen-gas etching^16^. Since the oxygen gas was not used during the process, the O atoms of the SiC/Si_4_O_5_N_3_ structure came from the residual oxygen in the hydrogen or nitrogen gases.

In the case of the GaN/Si_4_O_5_N_3_ structure, we need to supply Si atoms, for GaN has no Si atoms. We therefore propose that the GaN/Si_4_O_5_N_3_ structure can be synthesized by a two-step method as follows:

1. Perform SiCl_4_-plasma etching of GaN^25^. As a result, some Si atoms are expected to be adsorbed on the surface of GaN.
2. Perform annealing under nitrogen ambient^26^, and then N atoms from the nitrogen gas, the adsorbed Si atoms, and residual O atoms are expected to arrange themselves to form the epitaxial Si_4_O_5_N_3_ layer on GaN.

### Electronic structure

**Figure 2:**
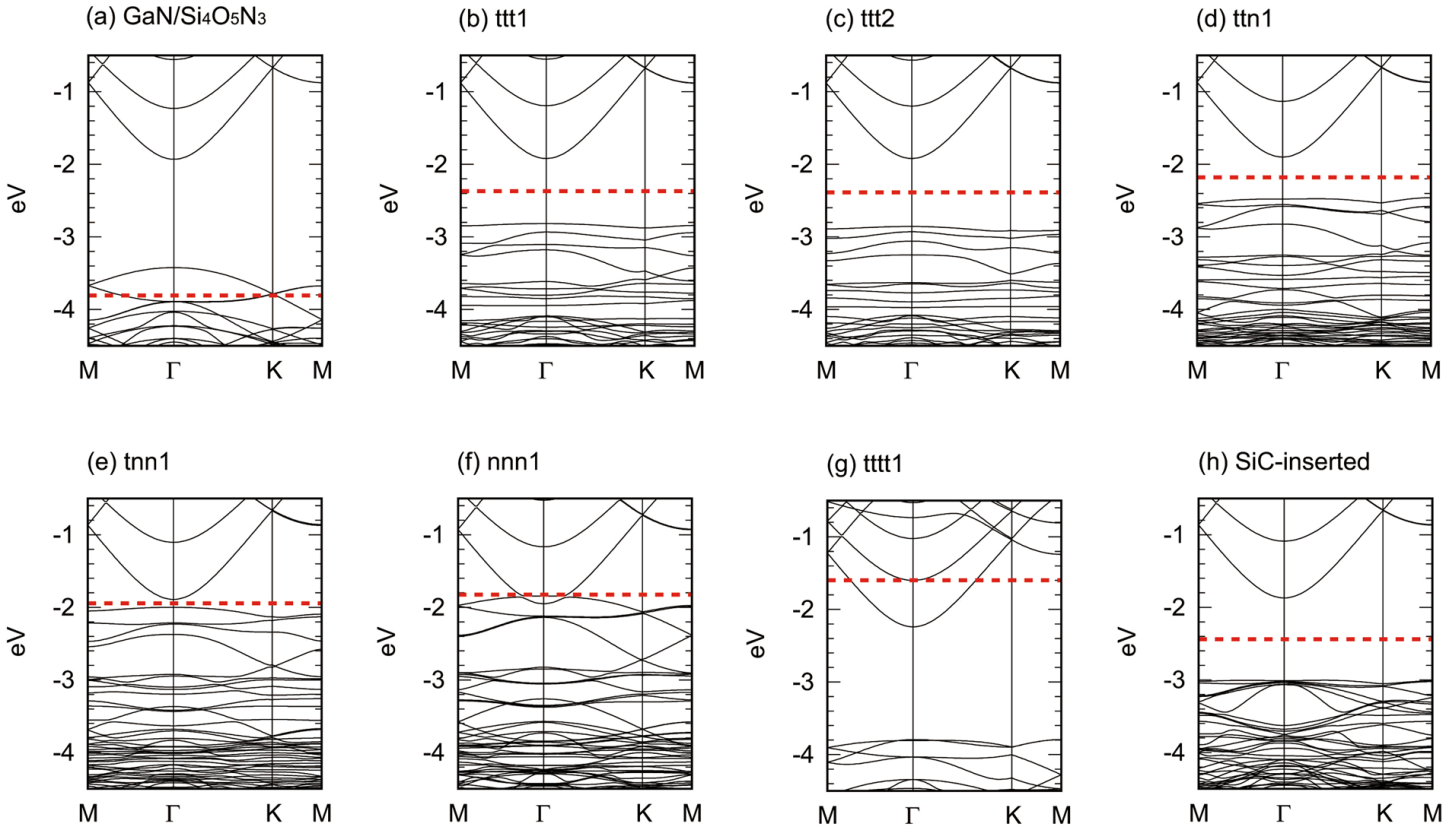
Band structure. (**a**) GaN/Si_4_O_5_N_3_ structure. (**b**) ttt1 structure. (**c**) ttt2 structure. (**d**) ttn1 structure. (**e**) tnn1 structure. (**f**) nnn1 structure. (**g**) tttt1 structure. (**h**) SiC-inserted structure. Each dashed line indicates the Fermi level, and the levels below the dashed line are filled by electrons. Note that the electrostatic potential is set to zero at infinity in the $[000\bar{1}]$ direction.

The band structure of the GaN/Si_4_O_5_N_3_ structure is shown in Fig. 2a. Since its valence band is partially empty, the GaN/Si_4_O_5_N_3_ structure is a degenerate p-type semiconductor. The partially empty valence band is explained by using the electron counting rules as follows^27^. The dangling bond of each Ga atom at the clean GaN(0001) surface contains 0.75 electrons, while the dangling bond of each N atom of the isolated Si_4_O_5_N_3_ layer contains one electron. When the Si_4_O_5_N_3_ layer is attached to the GaN(0001) surface, each pair of the interface Ga and N atoms forms a covalent bond, which we call an interface Ga-N bond. Unlike a Ga-N bond in GaN, each interface Ga-N bond is occupied by just 1.75 electrons, being short of 0.25 electrons to fulfil itself. Since there are total of twelve interface Ga-N bonds, it needs three more electrons per surface supercell to fulfil the valence band.

According to the rigid band model, since the valency of Si is larger than that of Ga by one, substituting three Ga atoms by three Si atoms makes the GaN/Si_4_O_5_N_3_ structure a semiconductor. We now examine this possibility. As substitution sites, we consider the Ga atoms in the topmost and the next GaN bilayers. According to the location of the doped Si atoms, the substitution patterns can be classified into four groups, which we call “ttt”, “ttn”, “tnn”, and “nnn”. Here, for example, ttn means that two Ga atoms in the topmost GaN bilayer and one Ga atom in the next GaN bilayer are substituted by Si atoms. As for ttt, since all the 12 Ga atoms in the topmost GaN-bilayer are equivalent, we may arbitrarily choose one Ga atom as the first substitution site. Since 11 Ga atoms are left, there are 11 × 10/2 = 55 possible combinations for second and third substitution sites. Note that, although the 55 patterns include all the symmetrically different patterns, but have duplication of symmetrically equivalent patterns. Similarly, we consider 11 × 12 = 134 different patterns for ttn, 12 × 11/2 = 66 for tnn, and 2 × 55 = 110 for nnn. Note that the “2” of “2 × 55” comes from the fact that 12 Ga atoms of the next GaN-bilayer is classified into two groups: eight Ga atoms lie under the Si atoms of the Si_4_O_5_N_3_ layer, while four Ga atoms do not.

**Figure 3:**
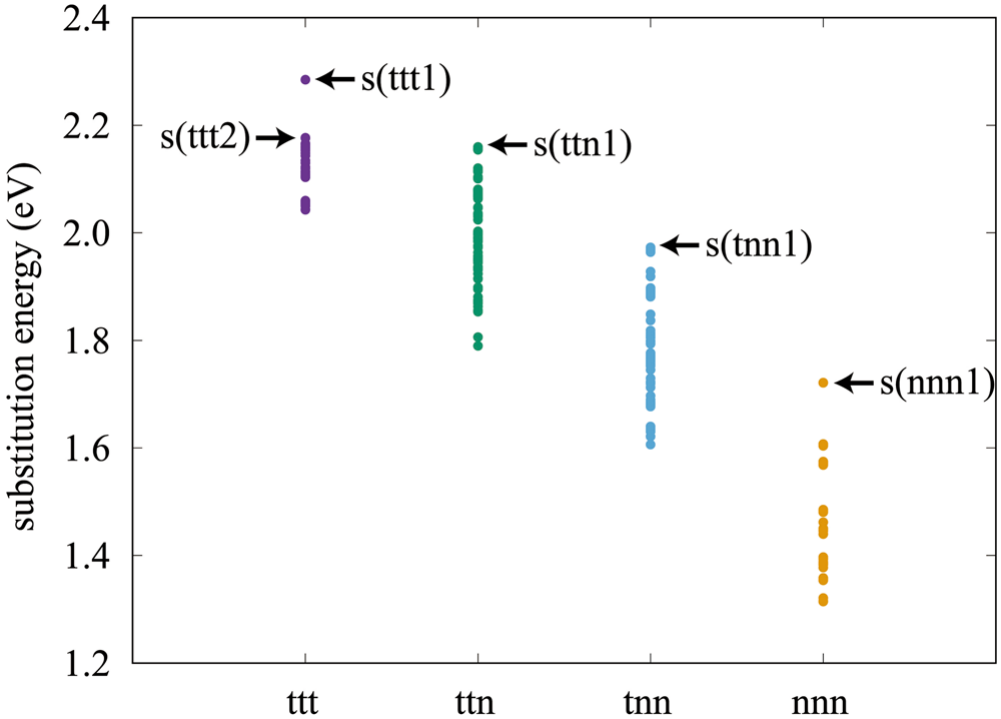
Substitution pattern dependence of substitution energy per Si atom. Each point indicates the substitution energy of a Si-doped GaN/Si_4_O_5_N_3_ structure.

**Figure 4:**
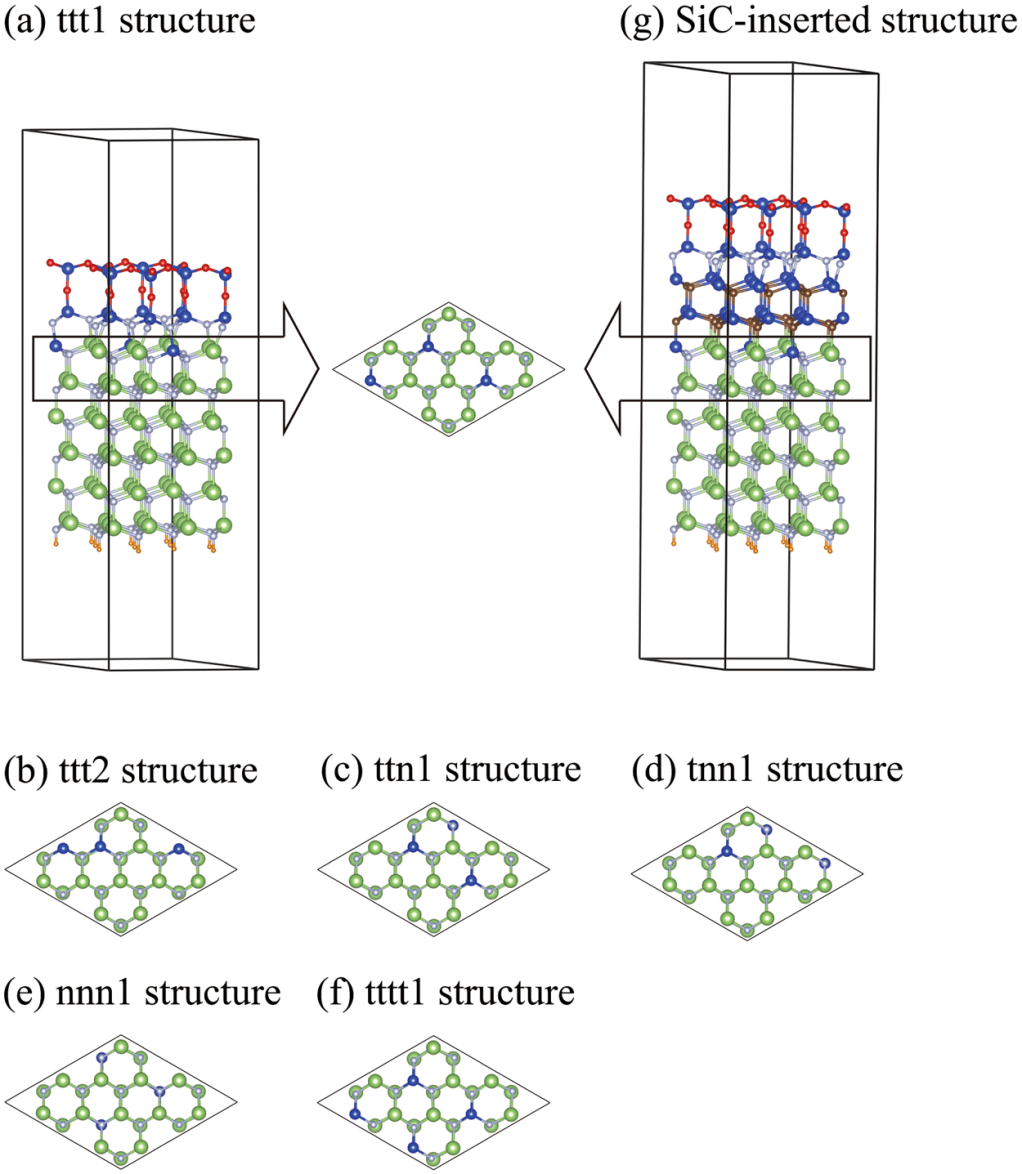
Structure model. (**a**) ttt1 structure. The arrow indicates the top view of the topmost and next GaN bilayers. (**b**) The topmost and next GaN bilayers of the ttt2 structure. (**c**) ttn1 structure. (**d**) tnn1 structure. (**e**) nnn1 structure. (**f**) tttt1 structure. (**g**) SiC-inserted structure. Brown spheres represent C atoms.

To determine the energetically most stable substitution pattern, we first optimized Si-doped structures using a lax convergence criterion of 1 × 10^−3^ Hartree/Bohr for forces on atoms, and calculated their substitution energies (Fig. 3). We then focused on ttt1, ttt2, ttn1, tnn1, and nnn1 structures (Fig. 4a–e), where the ttt2, for example, indicates a ttt structure whose substitution energy is the second highest in the ttt group. Note that, because of duplication of symmetrically equivalent patterns, three of 55 ttt structures have the highest energy. For reference, the substitution energies of the ttt1, ttt2, ttn1, tnn1, and nnn1 structures are 2.28, 2.18, 2.16, 1.97, and 1.72 eV/atom, respectively. We then optimized further the ttt1, ttt2, ttn1, tnn1, and nnn1 structures using a severe convergence criterion of 1 × 10^−4^ Hartree/Bohr, and refined their substitution energies. The refined substitution energies of the ttt1, ttt2, ttn1, tnn1, and nnn1 structures are 2.32, 2.19, 2.19, 1.99, and 1.73 eV/atom, respectively. From this result, we find that (1) the substitution doping is energetically favourable, (2) doped Si atoms prefer to locate the interface rather than the interior of GaN, and (3) the ttt1 structure is most stable.

To further study the stability of the ttt1 structure, we examine structures where four Si atoms are doped. Since doped Si atoms prefer to locate the interface, we consider only the tttt patterns. The tttt1 structure (energetically most stable structure in the tttt group) is illustrated in Fig. 4f. Its substitution energy is 1.89 eV/atom, and is smaller than that of the ttt1 structure. This suggests that Si atoms of the ttt1 structure do not aggregate to form the tttt1 structure.

The band structures of the ttt1, ttt2, ttn1, tnn1, nnn1 and tttt1 structures are shown in Fig. 2b–g. When three Si atoms are doped, the most stable ttt1 structure is a semiconductor. On the other hand, the tttt1 structure doped with four Si atoms is a degenerate n-type semiconductor (Fig. 2g), for the electrons are supplied to the conduction band. These results suggest that the type of the band structure can be tuned by the number of doped Si atoms which can be controlled by the amount of the adsorbed Si atoms.

**Figure 5:**
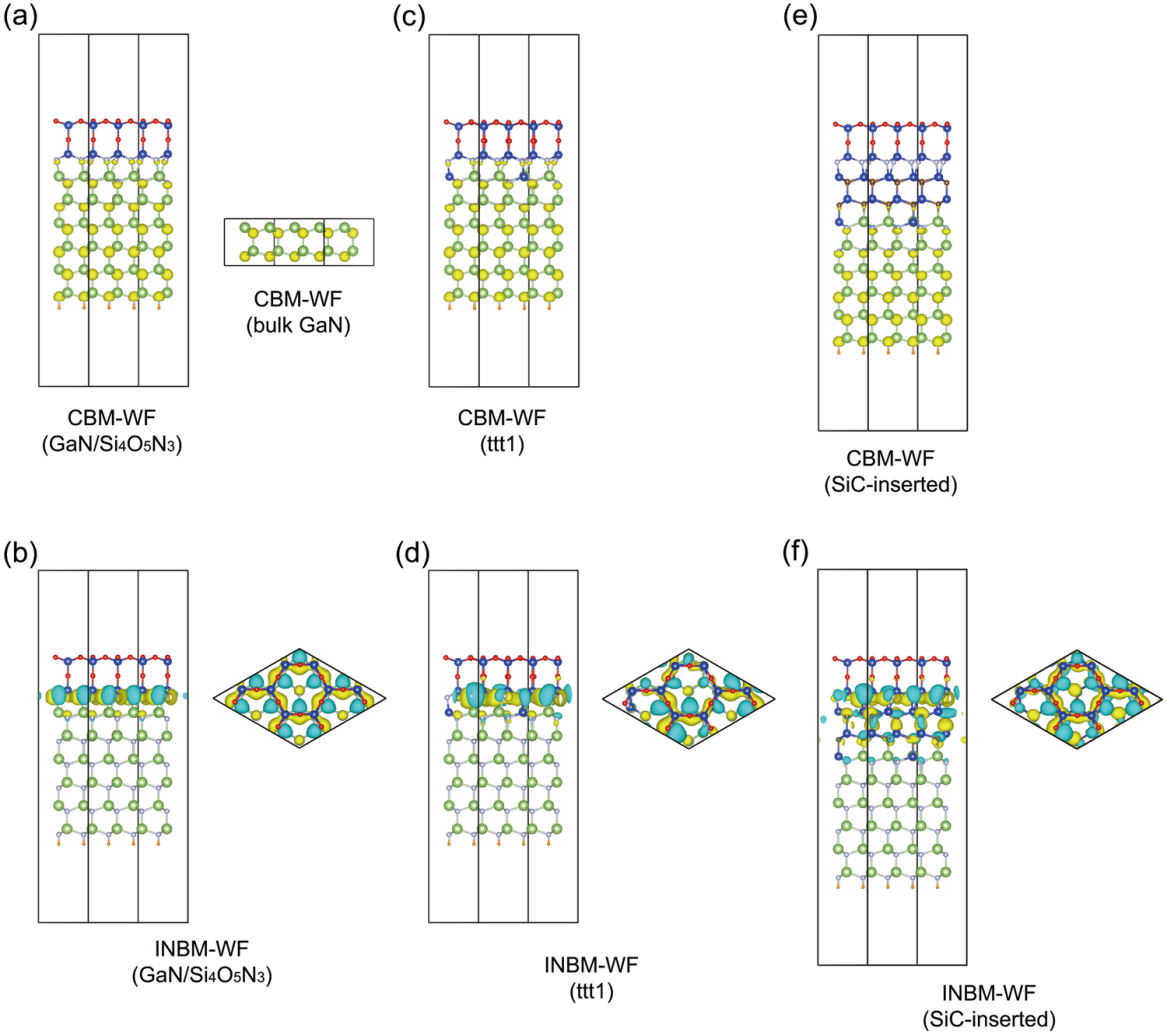
Isosurface of WF. (**a**) CBM-WFs of the GaN/Si_4_O_5_N_3_ structure and bulk GaN. (**b**) INBM-WF of the GaN/Si_4_O_5_N_3_ structure. (**c**) CBM-WF of the ttt1 structure. (**d**) INBM-WF of the ttt1 structure. (**e**) CBM-WF of the SiC-inserted structure. (**f**) INBM-WF of the SiC-inserted structure. The isovalue is ±0.015 $({{\rm{electron}}}^{1/2}/{{a}_{0}}^{3/2})$ except for bulk GaN where ±0.030 is used. Here, *a*_0_ is the Bohr radius.

The substitution pattern affects the band structure (Fig. 2b–f). Specifically, the band gap energies of the ttt1, ttt2, ttn1 and tnn1 structures are 0.90, 0.94, 0.58 and 0.11 eV, respectively. Even a metallic band structure is obtained when the Si atoms are doped in the nnn1 pattern (Fig. 2f). The substitution pattern dependence of the band gap energies can be explained in terms of the electrical double layer formed at the interface^28^. To show this, we first explain why the band gap energy of the ttt1 structure (0.90 eV) is smaller than that of the GaN/Si_4_O_5_N_3_ structure (1.50 eV). For this purpose, we first study the wave functions (WFs) of the GaN/Si_4_O_5_N_3_ structure. The conduction-band-minimum wave function (CBM-WF for short) consists of the *s* orbitals of the N atoms, resembling the CBM-WF of bulk GaN(Fig. 5a). On the other hand, the valence-band-maximum wave function (VBM-WF) of the GaN/Si_4_O_5_N_3_ structure consists of the ${p}_{\parallel }$ orbitals of the interface N atoms (Fig. 5b). Here, the ${p}_{\parallel }$ orbitals are the *p* orbitals pointing in directions parallel to the (0001) plane. We therefore refer to the VBM-WF as the interface-nitrogen band maximum wave function (INBM-WF for short). The CBM- and INBM-WFs of the ttt1 structure are essentially the same as those of the GaN/Si_4_O_5_N_3_ structure, respectively (Fig. 5c and d). Note that the INBM of the ttt1 structure is occupied, while that of the GaN/Si_4_O_5_N_3_ structure is unoccupied. Since electrons are transferred from the doped Si atoms to the interface N atoms to fulfil the interface-nitrogen band, an electrical double layer is formed at the interface of the ttt1 structure. As a result, an electrostatic potential difference is created between the Si_4_O_5_N_3_ layer and the GaN region. Because of the potential difference, the energy of the INBM of the ttt1 structure (−2.82 eV) is larger than that of the GaN/Si_4_O_5_N_3_ structure (−3.43 eV). On the other hand, since the CBM-WF is bulklike and consists of the atomic orbitals of the GaN region, the formation of the electrical double layer only minimally affects the energy of the CBM. In fact, the energy of the CBM of the ttt1 structure (−1.92 eV) is comparable to that of the GaN/Si_4_O_5_N_3_ structure (−1.93 eV). As a result, ${E}_{{\rm{g}}}^{{\rm{ttt}}1}={\varepsilon }_{{\rm{C}}}^{{\rm{ttt}}1}-{\varepsilon }_{{\rm{I}}}^{{\rm{ttt}}1}=0.90\,{\rm{eV}}$ is smaller than ${E}_{{\rm{g}}}^{{\rm{GaN}}/{{\rm{Si}}}_{4}{{\rm{O}}}_{5}{{\rm{N}}}_{3}}={\varepsilon }_{{\rm{C}}}^{{\rm{GaN}}/{{\rm{Si}}}_{4}{{\rm{O}}}_{5}{{\rm{N}}}_{3}}-{\varepsilon }_{{\rm{I}}}^{{\rm{ttt}}1}=1.50\,{\rm{eV}}$. Here, ${E}_{{\rm{g}}}^{{\rm{X}}}$, ${\varepsilon }_{{\rm{C}}}^{{\rm{X}}}$, and ${\varepsilon }_{{\rm{I}}}^{{\rm{X}}}$ are the band gap energy, the energy of the CBM, and the energy of the INBM of the structure X, respectively.

The potential difference increases as the distance between the electrical layers increases. Therefore, the energy of the INBM of the Si-doped GaN/Si_4_O_5_N_3_ structure increases when the Si atoms are doped farther from the interface N atoms. Accordingly, the band gap energy decreases as the number of Si atoms doped in the next bilayer increases: ${E}_{{\rm{g}}}^{{\rm{ttn}}1}=0.58\,{\rm{eV}}$ and ${E}_{{\rm{g}}}^{{\rm{tnn}}1}=0.11\,{\rm{eV}}$. The nnn1 structure is a metal, for ${\varepsilon }_{{\rm{I}}}^{{\rm{nnn}}1}=-1.84\,{\rm{eV}}$ becomes larger than ${\varepsilon }_{{\rm{C}}}^{{\rm{nnn}}1}=-1.95\,{\rm{eV}}$. Since the potential difference in the ttt2 structure is comparable to that in the ttt1 structure, ${E}_{{\rm{g}}}^{{\rm{ttt}}2}=0.94\,{\rm{eV}}$ is comparable to ${E}_{{\rm{g}}}^{{\rm{ttt}}1}=0.90\,{\rm{eV}}$. For reference, *E*_g_, *ε*_C_, and *ε*_I_ of the GaN/Si_4_O_5_N_3_, ttt1, ttt2, ttn1 and tnn1 structures are summarized in Table 1.

**Table 1:** Band gap energy (*E*_g_), energy of the bulklike CBM-MO band (*ε*_C_), and energy of the INBM-MO (*ε*_I_).

structure	*E*_g_	*ε*_C_	*ε*_I_
GaN/Si_4_O_5_N_3_	1.50	−1.93	−3.43
ttt1	0.90	−1.92	−2.82
ttt2	0.94	−1.92	−2.86
ttn1	0.58	−1.90	−2.48
tnn1	0.11	−1.89	−2.00
nnn1	0.00	−1.95	−1.84
SiC-inserted	1.14	−1.87	−3.01

The unit of energy is eV. Note that, for the GaN/Si_4_O_5_N_3_, ttt1, ttt2, ttn1, tnn1, and SiC-inserted structures, *E*_g_ = *ε*_C_ − *ε*_I_. Since *ε*_C_ < *ε*_I_, the nnn1 structure is a metal and *E*_g_ = 0.

The calculated band gap energy of the ttt1 structure (0.90 eV) is smaller than that of bulk GaN(1.84 eV). Note that the density functional theory underestimates the band gap energy and that the experimental value of bulk GaN is 3.42 eV^6^. Since a larger band gap energy is desirable for the application of GaN to power electronic devices, we attempt to increase the band gap energy. Given that the interface-nitrogen band is the origin of the narrow band gap, modifying the chemical components around the interface N atoms is a possible approach to enlarge the band gap. To examine this possibility, we point out that the interface-nitrogen band of the SiC/Si_4_O_5_N_3_ structure lies below its VBM^17^. In addition, since GaN can grow on a SiC substrate experimentally^6^, it would be experimentally possible to deposit SiC on a GaN substrate as well. We therefore examine a structure where two SiC bilayers (Si_24_C_24_) are inserted between the Si_4_O_5_N_3_ layer and the Si-doped GaN of the ttt1 structure (Fig. 4g).

**Figure 6:**
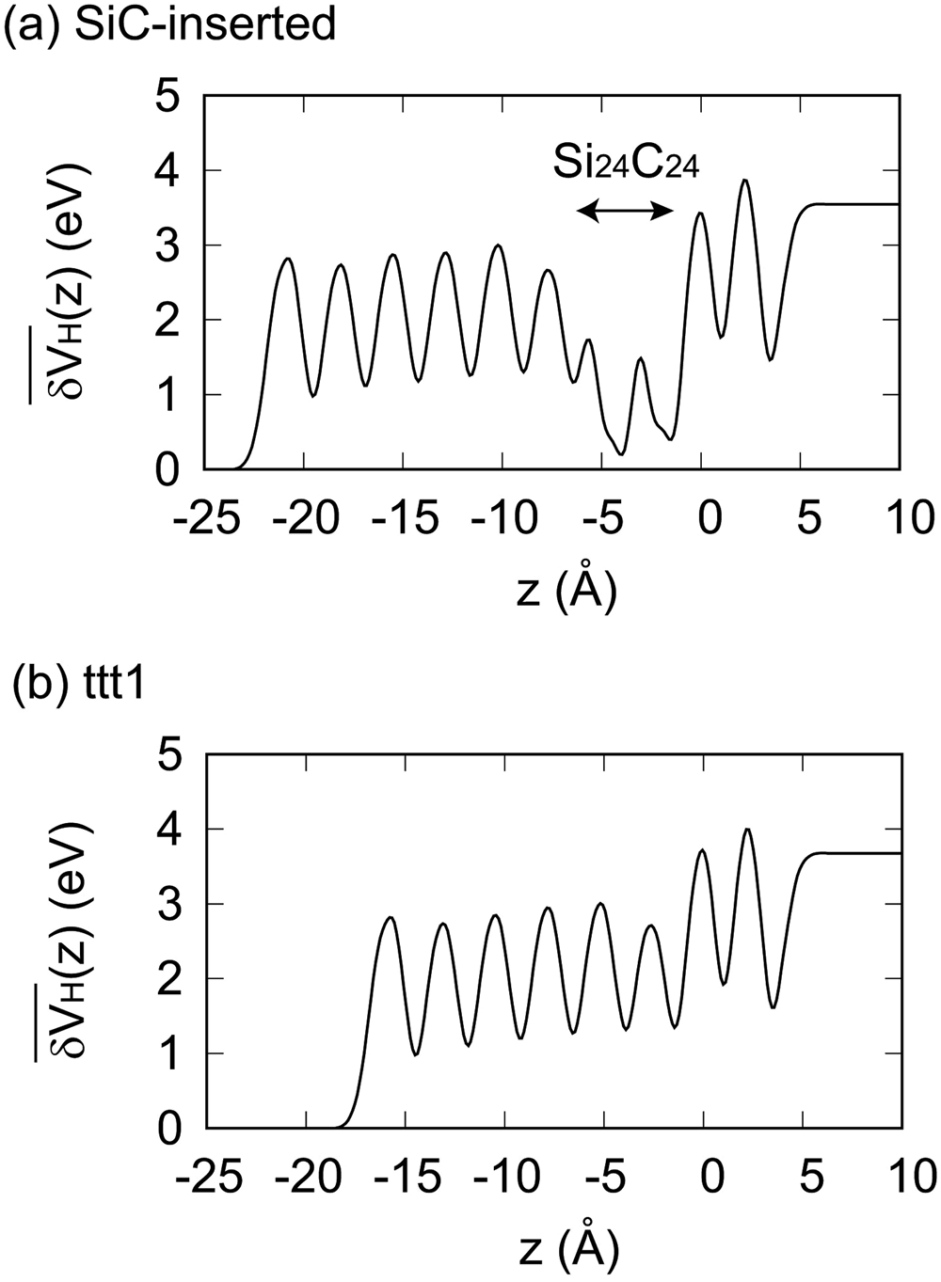
Profile of the average difference Hartree potential $\overline{\delta {V}_{H}}(z)$. (**a**) SiC-inserted structure. (**b**) ttt1 structure. The z axis is set to be along the [0001] direction. The average z coordinate of the interface N atoms is set to zero. Note that $\overline{{\rm{\delta }}{V}_{{\rm{H}}}}(-\infty )=0$.

The band gap energy of the SiC-inserted structure is 1.14 eV (Fig. 2h and Table 1). Although the band gap energy is still smaller than that of bulk GaN, tuning chemical environments around the interface N atoms is found to be effective for increasing the band gap energy. To explain why the band gap energy of the SiC-inserted structure is larger than that of the ttt1 structure, we study the profiles of the averaged difference Hartree potential^29^ $(\overline{{\rm{\delta }}{V}_{{\rm{H}}}}(z))$, which represents the electrostatic potential induced by charge transfer (See Methods for details). The averaged difference Hartree potential significantly drops in the SiC bilayers (Fig. 6a). On the other hand, such a drop is not found near the interface of the ttt1 structure (Fig. 6b). As with the ttt1 structure, the CBM-WF of the SiC-inserted structure mainly consists of the s orbitals of the N atoms. Therefore, the potential drop in the SiC bilayers does not affect the energy of the CBM. Therefore, ${\varepsilon }_{{\rm{C}}}^{{\rm{SiC}}-{\rm{inserted}}}=-1.87\,{\rm{eV}}$ is comparable to ${\varepsilon }_{{\rm{C}}}^{{\rm{ttt}}1}=-1.92\,{\rm{eV}}$ (Table 1). On the other hand, although the INBM-WF of the SiC-inserted structure resembles that of the ttt1 structure, the atomic orbitals of the SiC bilayers are mixed in it (Fig. 5f). Since the electrons in the SiC bilayers contribute to decrease the electrostatic potential energy of the INBM, ${\varepsilon }_{{\rm{I}}}^{{\rm{SiC}}-{\rm{inserted}}}=-3.01\,{\rm{eV}}$ is smaller than ${\varepsilon }_{{\rm{I}}}^{{\rm{ttt}}1}=-2.82\,{\rm{eV}}$ (Table 1). As a result, the band gap energy of the SiC-inserted structure is larger than that of the ttt1 structure.

Finally, we note that there are pros and cons to both the ttt1 and SiC-inserted structures. The latter is more advantageous for power electric devices than the former in terms of the band gap energy. On the other hand, because of its structural complexity, synthesizing the SiC-inserted structure may be more difficult than the ttt1 structure.

## Conclusion

We have proposed that the Si_4_O_5_N_3_ layer can epitaxially grow on a GaN(0001) surface without creating interface dangling bonds. Our *ab initio* calculations have shown that the dangling-bond-free GaN/Si_4_O_5_N_3_ structure is energetically more stable than the GaN/Si_2_O_5_ and GaN/Si_2_O_3_ structures. The electronic properties of the GaN/Si_4_O_5_N_3_ structure can be tuned by modifying the chemical components near the interface N atoms. We have also proposed a possible approach to experimentally synthesize the GaN/Si_4_O_5_N_3_ structure. Since it can be used as a seed for a high-quality GaN/SiO_2_ interface, the demonstration of the GaN/Si_4_O_5_N_3_ structure would be a milestone for improving the performance of GaN-based MISFETs.

## Methods

### Ab initio calculation

The *ab initio* calculations were performed using the OpenMX code^30^ which is based on the density functional theory with the generalized gradient approximation^31^ and the norm-conserving pseudopotentials^32^. The wave functions were expressed by the linear combination of pseudo-atomic orbitals (LCAO). The basis sets, Ga7.0-s2p2d2, N5.0s2p2d1, Si7.0-s2p2d1, O5.0-s2p2d1, PH5.0-s2p1, and C5.0-s2p2d1, were used for Ga, N, Si, O, pseudohydrogen with a fractional valence of 0.75^33–35^ and C atoms, respectively. Here, the abbreviation, for example Ga7.0-s2p2d2, indicates that two *s*, two *p*, and two *d* orbitals of a Ga atom with the cutoff radius of 7.0 Bohr were employed. Note that it was reported that the poor minimal basis set (Si7.0-s1p1 and C5.0-s1p1) did not reproduce the conduction band edge state of SiC^36^. However, we have confirmed that the proper basis set (Si7.0-s2p2d1 and C5.0-s2p2d1) reproduces it (Supplementary Fig. S1).

To study the Si_4_O_5_N_3_ layer on a GaN(0001) surface, a slab model composed of six GaN bilayers was used (Fig. 1a). Note that GDIS^37^ was used to construct the structure models and VESTA^38^ was used to illustrate the structure models. The effective screening medium method was employed to simulate an isolated slab^39,40^. The electrostatic potential is set zero at infinity in the $[000\bar{1}]$ direction. The dangling bonds of N atoms at the artificial GaN($000\bar{1}$) surface are terminated by pseudohydrogen atoms with a fractional valence of 0.75^33–35^. The two GaN bilayers adjoining to the pseudohydrogen atoms were fixed in optimizing the geometry of the slab model. The $(\sqrt{3}\times \sqrt{3})R{30}^{^\circ }$ surface supercell is the primitive one for the GaN/Si_4_O_5_N_3_ structure. However, to study the substitutional doping effects on the band structure, we used the $(2\sqrt{3}\times 2\sqrt{3})R{30}^{^\circ }$ surface supercell, except for molecular dynamics simulations where the $(4\sqrt{3}\times 4\sqrt{3})R{30}^{^\circ }$ surface supercell was used. The in-plane unit-cell vectors were constrained to match the bulk GaN(0001) surface. In the geometry optimizations, the convergence criterion of 1 × 10^−4^ Hartree/Bohr for forces on atoms was used unless noted otherwise. The 2 × 2 × 1 mesh of *k* points was used in the geometry optimizations. The 1 × 1 × 1 mesh of *k* points was used in the molecular dynamics simulations. The 11 × 11 × 1 mesh of *k* points was used in the band structure calculations.

### Formation energy

The formation energy of the GaN/Si_4_O_5_N_3_ structure per Si_2_N_3_ relative to the GaN/Si_2_O_5_ structure is defined as

1 $${f}_{{\rm{GaN}}/{{\rm{Si}}}_{4}{{\rm{O}}}_{5}{{\rm{N}}}_{3}}=-({E}_{{\rm{GaN}}/{{\rm{Si}}}_{4}{{\rm{O}}}_{5}{{\rm{N}}}_{3}}-12{\mu }_{{\rm{N}}}-8{\mu }_{{\rm{Si}}}-{E}_{{\rm{GaN}}/{{\rm{Si}}}_{2}{{\rm{O}}}_{5}})/4,$$

where ${E}_{{\rm{GaN}}/{{\rm{Si}}}_{4}{{\rm{O}}}_{5}{{\rm{N}}}_{3}}$ is the total energy of the GaN/Si_4_O_5_N_3_ per supercell, *μ*_N_ and *μ*_Si_ correspond to the energies per atom calculated for an N_2_ molecule and bulk Si, respectively, and ${E}_{{\rm{GaN}}/{{\rm{Si}}}_{2}{{\rm{O}}}_{5}}$ is the total energy of the GaN/Si_2_O_5_ structure per supercell. Note that the GaN/Si_2_O_5_ supercell is obtained by removing four Si_2_N_3_ units from the GaN/Si_4_O_5_N_3_ supercell, and the number of N (Si) atoms in the GaN/Si_4_O_5_N_3_ supercell is larger than that in the GaN/Si_2_O_5_ supercell by 12 (8).

We also define the formation energy of the GaN/Si_4_O_5_N_3_ structure per N atom relative to the GaN/Si_2_O_5_ structure as

2 $${f}_{{\rm{GaN}}/{{\rm{Si}}}_{4}{{\rm{O}}}_{5}{{\rm{N}}}_{3}}^{\ast }=-({E}_{{\rm{GaN}}/{{\rm{Si}}}_{4}{{\rm{O}}}_{5}{{\rm{N}}}_{3}}-12{\mu }_{{\rm{N}}}-8{\mu }_{{\rm{Si}}}-{E}_{{\rm{GaN}}/{{\rm{Si}}}_{2}{{\rm{O}}}_{5}})/12.$$

Note that $${f}_{{\rm{GaN}}/{{\rm{Si}}}_{4}{{\rm{O}}}_{5}{{\rm{N}}}_{3}}^{\ast }={f}_{{\rm{GaN}}/{{\rm{Si}}}_{4}{{\rm{O}}}_{5}{{\rm{N}}}_{3}}/3$$.

The formation energy of the GaN/Si_2_O_3_ structure per O atom relative to the GaN/Si_2_O_5_ structure is defined as

3 $${f}_{{\rm{GaN}}/{{\rm{Si}}}_{2}{{\rm{O}}}_{3}}=-({E}_{{\rm{GaN}}/{{\rm{Si}}}_{2}{{\rm{O}}}_{3}}+8{\mu }_{{\rm{O}}}-{E}_{{\rm{GaN}}/{{\rm{Si}}}_{2}{{\rm{O}}}_{5}})/8,$$

where ${E}_{{\rm{GaN}}/{{\rm{Si}}}_{2}{{\rm{O}}}_{3}}$ is the total energy of the GaN/Si_2_O_3_ per supercell, *μ*_o_ corresponds to the energy per atom calculated for an O molecule. Note that the number of O atoms in the GaN/Si_2_O_3_ supercell is smaller than that in the GaN/Si_2_O_5_ supercell by 8.

One may claim that LCAO methods cannot properly calculate the energies of molecules, hence the formation energies. To check the accuracy, we compared the formation energies of β-Si_3_N_4_ and α-SiO_2_ obtained using the OpenMX code with those obtained using the QMAS code^24,41^ which is based on the projector-augmented-wave method. Here, the formation energy of β-Si_3_N_4_ per N atom is defined as

4 $${f}_{{\rm{\beta }}-{{\rm{Si}}}_{3}{{\rm{N}}}_{4}}=-({E}_{{\rm{\beta }}-{{\rm{Si}}}_{3}{{\rm{N}}}_{4}}-3{\mu }_{{\rm{Si}}}-4{\mu }_{{\rm{N}}})/4,$$

where ${E}_{{\rm{\beta }}-{{\rm{Si}}}_{3}{{\rm{N}}}_{4}}$ is the total energy of β-Si_3_N_4_ per Si_3_N_4_. The formation energy of α-SiO_2_ per O atom is defined as

5 $${f}_{{\rm{\alpha }}-{{\rm{SiO}}}_{2}}=-({E}_{{\rm{\alpha }}-{{\rm{SiO}}}_{2}}-{\mu }_{{\rm{Si}}}-2{\mu }_{{\rm{O}}})/2,$$

where ${E}_{{\rm{\alpha }}-{{\rm{SiO}}}_{2}}$ is the total energy of α-SiO_2_ per SiO_2_. We found that the OpenMX code underestimates ${f}_{{\rm{\beta }}-{{\rm{Si}}}_{3}{{\rm{N}}}_{4}}$ by 0.01 eV and overestimates ${f}_{{\rm{\alpha }}-{{\rm{SiO}}}_{2}}$ by 0.06 eV. The errors are considerably small compared to ${f}_{{\rm{GaN}}/{{\rm{Si}}}_{4}{{\rm{O}}}_{5}{{\rm{N}}}_{3}}^{\ast }$= 5.80 eV and ${f}_{{\rm{GaN}}/{{\rm{Si}}}_{2}{{\rm{O}}}_{3}}$ = −3.95 eV.

### Substitution energy

The substitution energy per Si atom is defined as

6 $$s=-({E}_{\mathrm{Si}-\mathrm{doped}}-n{\mu }_{{\rm{Si}}}+n{\mu }_{{\rm{Ga}}}-{E}_{{\rm{GaN}}/{{\rm{Si}}}_{4}{{\rm{O}}}_{5}{{\rm{N}}}_{3}})/n,$$

where *E*_Si-doped_ is the total energy of the Si-doped GaN/Si_4_O_5_N_3_ structure per supercell, *n* is the number of doped Si atoms, and *μ*_Ga_ is the energy per atom calculated for bulk α-Ga.

### Average difference Hartree potential

The averaged difference Hartree potential is defined as

7 $$\overline{{\rm{\delta }}{V}_{{\rm{H}}}}(z)=\iint {\rm{\delta }}{V}_{H}(x,y,z)dxdy/\iint dxdy$$

where the z axis is set to be along the [0001] direction, δ*V*_H_(*x*, *y*, *z*) is the difference Hartree potential^29^ at (*x*, *y*, *z*) associated with the difference electron density $({\rm{\delta }}n({\boldsymbol{r}})=n({\boldsymbol{r}})-\sum _{i}{n}_{i}^{({\rm{a}})}({\boldsymbol{r}}))$, where *n*(***r***) is the electron density and ${n}_{i}^{({\rm{a}})}({\boldsymbol{r}})$ is the electron density of the atom *i*. Note that $\overline{{\rm{\delta }}{V}_{{\rm{H}}}}(-\infty )=0$.

## Electronic supplementary material

Supplemental Information

## References

[1] Tu Y, Tersoff J (2000). Structure and Energetics of the Si- SiO2 Interface. Phys. Rev. Lett..

[2] Ourmazd A, Taylor DW, Rentschler JA, Bevk J (1987). Si → SiO2 transformation: Interfacial structure and mechanism. Phys. Rev. Lett..

[3] Ikarashi N, Watanabe K, Miyamoto Y (2000). High-resolution transmission electron microscopy of an atomic structure at a Si(001) oxidation front. Phys. Rev. B.

[4] Kageshima H (2006). Theoretical Study on Atomic Structures of Thermally Grown Silicon Oxide/Silicon Interfaces. e-Journal of Surface Science and Nanotechnology.

[5] Placidi M (2010). Deposited Thin SiO2 for Gate Oxide on n-Type and p-Type GaN. J. Electrochem. Soc..

[6] Lidow, A., Strydom, J., Rooij, M. de & Reusch, D. *GaN Transistors for Efficient Power Conversion*, Ch. 1, 1–18 (Wiley, 2014).

[7] Gu S (2014). Characterization of interface and border traps in ALD Al2O3/GaN MOS capacitors with two-step surface pretreatments on Ga-polar GaN. Applied Surface Science.

[8] Eller BS, Yang J, Nemanich RJ (2013). Electronic surface and dielectric interface states on GaN and AlGaN. Journal of Vacuum Science & Technology A.

[9] Arulkumaran S, Egawa T, Ishikawa H, Jimbo T, Umeno M (1998). Investigations of SiO2/n-GaN and Si3N4/n-GaN insulator–semiconductor interfaces with low interface state density. Applied Physics Letters.

[10] Yamaji K, Noborio M, Suda J, Kimoto T (2008). Improvement of Channel Mobility in Inversion-Type n-Channel GaN Metal–Oxide–Semiconductor Field-Effect Transistor by High-Temperature Annealing. Japanese Journal of Applied Physics.

[11] Jr HCC, Fountain GG, Alley RG, Keller BP, DenBaars SP (1996). Low interface trap density for remote plasma deposited SiO2 on n‐type GaN. Applied Physics Letters.

[12] Mitsuishi K (2017). Electron microscopy studies of the intermediate layers at the SiO2/GaN interface. Jpn. J. Appl. Phys..

[13] Bernhardt J, Schardt J, Starke U, Heinz K (1999). Epitaxially ideal oxide–semiconductor interfaces: Silicate adlayers on hexagonal (0001) and (0001̄) SiC surfaces. Appl. Phys. Lett..

[14] Hollering M (1999). Electronic states of an ordered oxide on C-terminated 6H–SiC. Surface Science.

[15] Lu W, Krüger P, Pollmann J (2000). Atomic and electronic structure of silicate adlayers on polar hexagonal SiC surfaces. Phys. Rev. B.

[16] Shirasawa T (2007). Epitaxial Silicon Oxynitride Layer on a 6H-SiC(0001) Surface. Phys. Rev. Lett..

[17] Krüger P, Baumeier B, Pollmann J (2008). First-principles investigation of an epitaxial silicon oxynitride layer on a 6H-SiC(0001) surface. Phys. Rev. B.

[18] Devynck F, Šljivančanin Ž, Pasquarello A (2007). Electronic properties of an epitaxial silicon oxynitride layer on a 6H-SiC(0001) surface: A first-principles investigation. Applied Physics Letters.

[19] Ando Y, Gohda Y, Tsuneyuki S (2012). Dependence of the Schottky barrier on the work function at metal/SiON/SiC(0001) interfaces identified by first-principles calculations. Surface Science.

[20] Tochihara H, Shirasawa T (2011). The epitaxial crystalline silicon-oxynitride layer on SiC(0001): Formation of an ideal SiC–insulator interface. Progress in Surface Science.

[21] Takeda K, Shiraishi K (1994). Theoretical possibility of stage corrugation in Si and Ge analogs of graphite. Phys. Rev. B.

[22] Vogt P (2012). Silicene: Compelling Experimental Evidence for Graphenelike Two-Dimensional Silicon. Phys. Rev. Lett..

[23] Fleurence A (2012). Experimental Evidence for Epitaxial Silicene on Diboride Thin Films. Phys. Rev. Lett..

[24] Nishio K, Lu AKA, Pourtois G (2015). Low-strain Si/O superlattices with tunable electronic properties: Ab initio calculations. Phys. Rev. B.

[25] Zhang L (1996). Electron cyclotron resonance etching characteristics of GaN in SiCl4/Ar. Appl. Phys. Lett..

[26] Zolper JC, Hagerott Crawford M, Howard AJ, Ramer J, Hersee SD (1996). Morphology and photoluminescence improvements from high‐temperature rapid thermal annealing of GaN. Appl. Phys. Lett..

[27] Pashley MD (1989). Electron counting model and its application to island structures on molecular-beam epitaxy grown GaAs(001) and ZnSe(001). Phys. Rev. B.

[28] Harrison WA, Kraut EA, Waldrop JR, Grant RW (1978). Polar heterojunction interfaces. Phys. Rev. B.

[29] Ozaki T, Kino H (2005). Efficient projector expansion for the ab initio LCAO method. Phys. Rev. B.

[30] Ozaki, T. *et al*. OpenMX website. http://www.openmx-square.org/.

[31] Perdew JP, Burke K, Ernzerhof M (1996). Generalized Gradient Approximation Made Simple. Phys. Rev. Lett..

[32] Morrison I, Bylander DM, Kleinman L (1993). Nonlocal Hermitian norm-conserving Vanderbilt pseudopotential. Phys. Rev. B.

[33] Shiraishi K (1990). A New Slab Model Approach for Electronic Structure Calculation of Polar Semiconductor Surface. J. Phys. Soc. Jpn..

[34] Pignedoli CA, Di Felice R, Bertoni CM (2001). Dissociative chemisorption of NH3 molecules on GaN(0001) surfaces. Phys. Rev. B.

[35] Rosa AL, Neugebauer J (2006). First-principles calculations of the structural and electronic properties of clean GaN(0001) surfaces. Phys. Rev. B.

[36] Matsushita Y, Furuya S, Oshiyama A (2012). Floating Electron States in Covalent Semiconductors. Phys. Rev. Lett..

[37] Fleming, S. The GDIS Home Page. http://gdis.seul.org/.

[38] Momma K, Izumi F (2011). *VESTA 3* for three-dimensional visualization of crystal, volumetric and morphology data. Journal of Applied Crystallography.

[39] Otani M, Sugino O (2006). First-principles calculations of charged surfaces and interfaces: A plane-wave nonrepeated slab approach. Phys. Rev. B.

[40] Ohwaki T, Otani M, Ikeshoji T, Ozaki T (2012). Large-scale first-principles molecular dynamics for electrochemical systems with O(N) methods. The Journal of Chemical Physics.

[41] Ishibashi, S. *et al*. QMAS | Quantum MAterials Simulator Official Site. http://qmas.jp/pub/index.html.

