# Supplementary material for: Ultrathin silicon oxynitride layer on GaN for dangling-bond-free GaN/insulator interface
Source: Sci Rep. 2018 Jan 23;8:1391. doi: 10.1038/s41598-018-19283-4 (PMC5780416; doi:10.1038/s41598-018-19283-4)
Supplement: Supplementary file 1 — Supplemental Information [file 41598_2018_19283_MOESM1_ESM.pdf]

Supplemental Information for "Ultrathin silicon oxynitride layer on GaN for dangling-bond-free GaN/insulator interface"

Kengo Nishio, Tomoe Yayama, Takehide Miyazaki, Noriyuki Taoka, and Mitsuaki Shimizu

Correspondence should be addressed to K. N. (k-nishio@aist.go.jp).

Contents

Supplementary Figure S1.

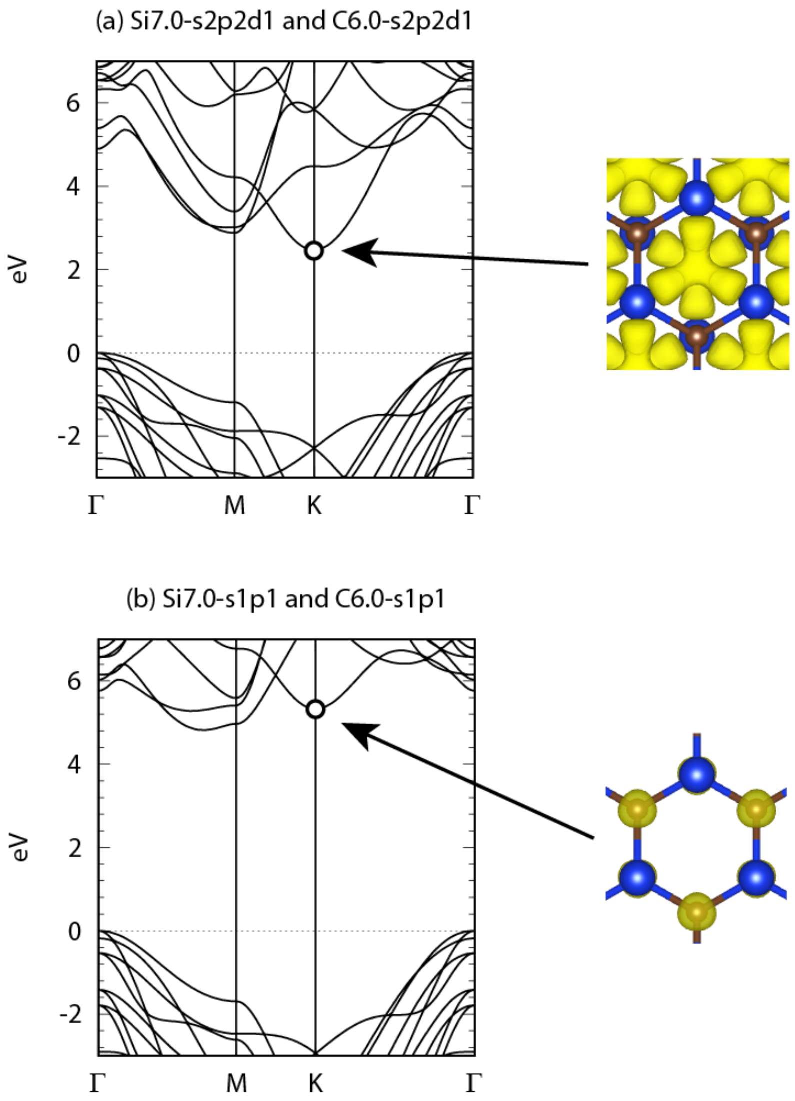

Supplementary Figure S2 | Basis set dependence of the band structure of 2H-SiC. **(a)** Band structure calculated by using the proper basis set (Si7.0-s2p2d1 and C5.0-s2p2d1). The isosurface of the squared molecular orbital of the arrowed level corresponding to 70% of the maximum value is also shown. **(b)** Band structure calculated by using the poor minimum basis set (Si7.0-s1p1 and C5.0-s1p1). The isosurface of the squared molecular orbital of the arrowed level corresponding to 40% of the maximum value is also shown. The conduction band minimum of 2H-SiC is properly located at K point when the proper basis set is used. On the other hand, the poor minimal basis set fails to reproduce it[36].
